# Acute Effects of Different Multivitamin Mineral Preparations with and without Guaraná on Mood, Cognitive Performance and Functional Brain Activation

**DOI:** 10.3390/nu5093589

**Published:** 2013-09-13

**Authors:** Andrew Scholey, Isabelle Bauer, Chris Neale, Karen Savage, David Camfield, David White, Silvia Maggini, Andrew Pipingas, Con Stough, Matthew Hughes

**Affiliations:** 1 Centre for Human Psychopharmacology, Swinburne University, Melbourne VIC 3122, Australia; E-Mails: ibauer@swin.edu.au (I.B.); chrisneale02@gmail.com (C.N.); karenmsavage@gmail.com (K.S.); david.camfield@gmail.com (D.C.); dawhite@swin.edu.au (D.W.); apipingas@swin.edu.au (A.P.); cstough@gmail.com (C.S.); matthewhughes@swin.edu.au (M.H.); 2 Bayer Consumer Care Ltd., Basel 4002, Switzerland; E-Mail: silvia.maggini@bayer.com

**Keywords:** multivitamin, guaraná, attention, working memory, mood, fMRI, neuroimaging

## Abstract

Previous work has identified the positive effects of the acute administration of a multivitamin-guaraná preparation during an effortful executive/working memory task. Here, we aimed to differentiate the effects of multivitamins with and without guaraná and to examine the neural substrates of such effects using functional magnetic resonance imaging (fMRI). Following a double-blind, placebo-controlled, randomised, balanced crossover design, 20 participants (mean age 29 ± 5.54 years) consumed multivitamin preparations with or without guaraná (Berocca^®^ Performance and Boost, respectively) and a placebo. Thirty minutes post-treatment, they underwent neurocognitive assessment, consisting of a 10 min Cognitive Demand Battery, with mood ratings taken immediately before and after the battery. Five additional participants underwent post-treatment fMRI scanning during Rapid Visual Information Processing and Inspection Time activation tasks. The multivitamin with guaraná treatment was associated with significantly enhanced Serial Threes performance and self-rated contentment. fMRI revealed that both multivitamin treatments increased activation in areas associated with working memory and attentional processing, with the effect being greater in the multivitamin with guaraná condition. These data confirm the acute benefits of multivitamins with guaraná on mood and cognitive performance. Furthermore, they demonstrate for the first time increased brain activation from multivitamin preparations both with and without guaraná, as measured using fMRI.

## 1. Introduction

It has been recognised for decades that micronutrient status can influence cognitive function across the lifespan, including that insufficient vitamin intake can impair several aspects of cognitive functions and mood [1,2,3,4,5,6,7,8]. In particular, the dietary status of certain B vitamins (e.g., folate, vitamins B6 and B12) has been positively associated with aspects of cognition, such as information processing, recall and verbal ability in healthy women of various ages [9]. A continuous supply of water-soluble vitamins is necessary to avoid deficiency, and even in otherwise healthy adults, water-soluble vitamin deficiency results in fatigue, anxiety, irritability, sleeplessness and impaired memory and ability to concentrate [10,11].

In recent years, there has been increasing interest in the possible modulating effects of multivitamin supplementation on mood and cognition (for recent reviews, see [12,13]). In cases of marginal or more severe deficiency, multivitamin supplementation has been shown to enhance cognitive functions [3]. More recent evidence suggests that such supplementation can improve behavioural function (*i.e.*, mood and cognitive functions/performance) even in the absence of vitamin deficiency. One study showed that 33-day multivitamin supplementation improved mood and attentional performance in males [14,15]. In the same study, perceived stress was reduced in the active treatment group, which is consistent with the findings of several other trials. For example, multivitamin supplementation reduced scores on the Depression, Anxiety and Stress scale in an eight-week study (DASS [16]), reduced occupational stress in a 90-day trial [17] and ameliorated negative mood in response to an acute laboratory stressor following a nine-week supplementation regimen [18].

As well as positive effects following chronic supplementation, some studies have raised the possibility that vitamin administration may improve behavioural outcomes acutely. For example, children’s attentional performance was improved in 3 h following administration of a multivitamin [19]. Of particular relevance to the current study is our previous report of acute improvements in working memory/vigilance performance and reduced mental fatigue following administration of a commercially available multivitamin and mineral preparation containing B vitamins and guaraná (Berocca^®^ Boost) [20]. While this may support the notion of acute effects from multivitamin supplementation, it is worth noting that the findings may be linked to the guaraná contained in the preparation. Guaraná has known neurocognitive effects (see [21]), including increased alertness, calmness and memory performance [22]. To the authors’ knowledge, there is no published study that has evaluated the neurocognitive effects of multivitamin supplementations with and without guaraná.

The beneficial effects of vitamin supplementation on cognition have been associated with the involvement of B vitamins and minerals in a range of biological mechanisms during cellular metabolism [23]. These include the positive effects of B vitamins on the synthesis and integrity of phospholipids, proteins and DNA [23]. There is also evidence that B vitamins are neuroprotective and lower homocysteine levels [24]. Furthermore, it has been suggested that the production of catecholamines and other monoamines is enhanced by increased B vitamin levels [14,23]. B vitamins are also involved in folate metabolism, and folate levels correlate with mood and cognitive performance [13]. While most research in this area has assumed that any effects of multivitamins are restricted to chronic supplementation, there are also reports of acute benefits to cognition from multivitamins, e.g., [19].

When attempting to evaluate the overall effects of nutritional supplementation on brain function, it is useful to collect measures of both cognition and neural activity. Functional magnetic resonance imaging (fMRI) techniques combined with in-scanner testing are ideal, as they enable researchers to simultaneously gather behavioural data and sample brain activity with a high degree of spatial resolution [25]. To our knowledge, no study to date has examined the effects of vitamins on brain activation using fMRI. 

In this study, we explored the effects of acute supplementation with multivitamin and mineral preparations, both with and without guaraná, on mood, cognition and fMRI measures. The cognitive assessment involved a single cycle of the Cognitive Demand Battery previously shown to be sensitive to the multivitamin with guaraná preparation [20]. Regarding fMRI, we specifically investigated functional brain activation during higher order cognitive functions by using Rapid Visual Information Processing (RVIP) and Inspection Time (IT) tasks. These tasks assess different stages of information processing and attention. The RVIP task is a serial presentation task, where participants are required to identify a target of three odd or three even numbers in a rapidly presented number stream. The IT task assesses early information processing in a forced choice, two-alternative test, where participants must discriminate the longer of two lines, displayed for a variable duration, prior to the onset of a backwards mask. Both tasks have previously been used in the scanner environment in healthy adults [26,27].

The primary aim of this study was to determine whether the administration of multivitamin preparations both with and without guaraná would differentially affect mood and mental performance when compared with a placebo. We hypothesised that the multivitamin and guaraná treatment would be associated with an improvement in mood and mental performance. This acted as a positive control against which to test the hypothesis that multivitamins alone would similarly improve mood and cognition. Additionally, fMRI was employed in a separate exploratory study to determine the effects of both treatments on the functional activation of the brain during higher order cognitive tasks.

## 2. Method

### 2.1. Design

The study was comprised of a behavioural trial and an fMRI experiment. Both employed randomised, double-blind, balanced, placebo-controlled, crossover designs. The study was approved by the Swinburne University Human Research Ethics Committee (Ref SUHREC 2010/300) and was conducted in accordance with the Declaration of Helsinki. All participants provided written informed consent. The trial was registered as ACTRN12612001116819.

### 2.2. Participants

Twenty healthy adults (8 male, 12 female) aged 21–39 years (mean age = 28.35 years, SD = 5.52) were recruited for the study from the Centre for Human Psychopharmacology participant database and via local media advertising. An additional five participants (mean age = 28.4 years, SD = 4.72) took part in the fMRI investigation. All subjects were healthy, non-smoking, right-handed, regular caffeine consumers. They met standard inclusion criteria, including being healthy, having no history of psychiatric or neurological illness and not taking over-the-counter or prescription medicine.

### 2.3. Treatments

Active treatments were two commercially available multivitamin and mineral effervescent tablets, Berocca^®^ Performance and Berocca Boost. The constituents are shown in Table 1. Compared with Berocca Performance, Berocca Boost also contains caffeine (40 mg) derived from guaraná (222 mg) and generally lower levels of B vitamins and vitamin C. These treatments were dissolved in 330 mL water and administered in unlabelled and coded bottles containing 330 mL of solution. The placebo was a 330 mL effervescent drink with similar colouring. To further optimise blinding, treatment bottles were placed in identical opaque sleeves before consumption to prevent sight of the liquids. 

**Table 1:** Contents of the vitamin/mineral tablets Berocca Boost and Berocca Performance effervescent tablets.

	Berocca Boost	Berocca Performance
**Vitamin B1**	1.40 mg	15 mg
**Riboflavin (Vitamin B2)**	1.60 mg	15 mg
**Nicotinamide (B3/niacin)**	18 mg	50 mg
**Vitamin B5**	6 mg	23 mg
**Vitamin B6**	2 mg	10 mg
**Folic Acid (Vitamin B9)**	200 μg	400 μg
**Vitamin B12**	1 μg	10 μg
**Biotin (Vitamin B7)**	150 μg	150 μg
**Vitamin C**	60 mg	500 mg
**Calcium**	100 mg	100 mg
**Magnesium**	100 mg	100 mg
**Zinc**	9.5 mg	10 mg
**Guaraná**	222.2 mg (40 mg of caffeine)	

### 2.4. Procedure

Participants in the behavioural part of the study attended the Centre for Human Psychopharmacology on four occasions (one practice visit and three study days). They were required to fast and refrain from drinking alcohol and caffeine for 12 h before testing sessions. During the first (practice) visit, participants were familiarised with the procedures, and morphometric and demographic data were recorded. This visit also served to familiarise subjects with task requirements and to attenuate any practice effects. On the three study days, participants provided baseline mood, stress and mental fatigue measures completed immediately before and after the computer-based Cognitive Demand Battery. They then consumed the treatment (which was made on the testing day by a disinterested third party). Exactly 30 min later, participants again completed mood, fatigue and stress questionnaires immediately before and after completion of a single 10-min cycle of the Cognitive Demand Battery. Following this, they underwent a steady-state topography (SST) recording (data from which will be presented elsewhere). Treatment order was determined by random allocation to a Latin square. 

### 2.5. Cognitive and Mood Measures

#### 2.5.1. Cognitive Demand Battery

The Cognitive Demand Battery involves completion of 2-min computerised versions of both Serial Threes (involving the repeated subtraction of three from a randomly generated starting number) and Serial Sevens (subtraction of seven) [28], followed by a 5-min version of the Rapid Visual Information Processing (RVIP) Task, during which participants monitor a continuous string of digits, presented at 100 per min, and respond when they detect three consecutive odd or even digits (there are eight correct targets per min). At the end of the 10 min battery, participants are asked to rate their “mental fatigue” on an analogue scale with the end-points marked “not at all” and “very much so”. Various forms of the Cognitive Demand Battery have been shown to be sensitive to the effects of nutritional interventions, including glucose alone [29] and with caffeine [30] and ginseng [29] to ginseng alone [31] and to cocoa polyphenols [32]. Of particular relevance to the current study, the multivitamin preparation with guaraná (Berocca Boost) improved performance on the Cognitive Demand Battery [20]. In the current study, participants underwent a single, 10-min cycle of the battery, as opposed to the 6 repetitions of Kennedy *et al.* (2008)’s study [20]. The reasons for this were twofold; firstly, to minimise the possibility of fatigue during subsequent SST imaging (reported elsewhere), and secondly, because in the Kennedy *et al*. study, increased performance and reduced fatigue associated with the guaraná-vitamin preparation were evident during the first cycle of the battery [20]. 

#### 2.5.2. State-Trait Anxiety Inventory [33]—State Portion (STAI-S)

Participants rated how much 20 statements matched their current mood (e.g., “I am calm”) by marking a four-point scale ranging from “not at all” to “very much so”. Scores range from 20 to 80, with higher scores indicating more anxiety. The “State” subscale is a widely used instrument for measuring fluctuating levels of anxiety.

#### 2.5.3. Visual Analogue Mood Scales (VAMS)

The Bond-Lader mood scales [34] comprise 16 × 100 mm visual analogue scales with the end points anchored by antonyms: alert-drowsy, calm-excited, strong-feeble, muzzy-clearheaded, well-coordinated-clumsy, lethargic-energetic, contented-discontented, troubled-tranquil, mentally slow-quick witted, tense-relaxed, attentive-dreamy, incompetent-proficient, happy-sad, antagonistic-friendly, interested-bored and withdrawn-sociable. Participants were presented with an A4 sheet containing all the scales and instructed to mark their current mood state on each line. These were combined as recommended by the authors to form three mood factors, “alert”, “calm” and “contented”, with scores on each ranging from 0 to 100. They also completed two additional 100 mm Visual Analogue Mood Scales (VAMS) evaluating “stress” and “fatigue” with the end-points marked “not at all” and “very much so”, respectively.

### 2.6. Functional Magnetic Imaging (fMRI) Measures

#### 2.6.1. fMRI Testing Procedure 

Five other participants underwent fMRI. They were scanned during three separate one-hour sessions (Placebo, Berocca Boost, Berocca Performance) at the Brain Research Institute (Austin Hospital, Heidelberg, Australia). Upon arrival at the MRI facility, the treatment was administered to the participant, who was allowed 5 min to consume the product. After 30 min of absorption, participants completed the STAI-S mood questionnaire and entered the scanner to perform the IT and RVIP tasks. After scanning, participants again completed the STAI-S mood paper questionnaire before debriefing and cessation of the session. All aspects of the tasks were controlled by Presentation^®^ software (Version 14.5). All stimuli was presented visually on a computer monitor and viewed via a mirror on the head coil. The eye-to-screen distance was about 4 m.

#### 2.6.2. Imaging Procedures

Participants were scanned using a 3 Tesla Siemens Tim Trio MRI scanner (Siemens, Erlangen, Germany) fitted with a 12 channel head-coil. In the first session, a high resolution T1 weighted image was acquired (coronal slice acquisition) using a 3D MPRAGE sequence; time-to-repeat (TR) = 1900 ms, time-to-echo (TE) = 2.6 ms, 192 slices, 0.9 × 0.9 × 0.9 voxel, field of view (FOV) = 230 mm, slice thickness = 0.9 mm. In each of the subsequent testing sessions, functional images were acquired using a T2* weighted gradient echo-planar image (EPI) sequence (TR = 3000 ms, TE = 30 ms, FOV = 216 mm, voxel size = 3 × 3 × 3 mm) while participants performed the cognitive tasks. For the RVIP task, 145 volumes were collected per run, while 141 volumes were collected during each IT individual run. The commencement of the functional paradigm was triggered by the onset of the fourth volume, and volumes 1 through 3 were discarded to negate T1 saturation effects in EPI images. Each image was comprised of 44 3-mm slices acquired approximately axially in an ascending order. Participants were asked to minimise head movement, which was aided by foam padding inserted around the participant’s head and neck. Participants held an MRI-compatible button box in their right hand used to respond to the stimuli and were provided with a microphone to enable communication with the researcher and MRI technician while in the scanner. Stimuli were presented on an MRI-compatible screen positioned behind the scanner that participants viewed via a mirror mounted on the head-coil. All preprocessing and statistical analysis of image data was performed using SPM8 (Wellcome Trust Centre for Neuroimaging, London, UK) and associated toolboxes.

#### 2.6.3. fMRI Preprocessing

Initially, “ArtRepair” [35] routines were used to minimise voxel noise and repair aberrant image slices in EPIs. These corrected images were realigned to the first image in the first session, and a mean realigned image was created. The high resolution T1 weighted structural image was then co-registered to the mean realigned image. After visually inspecting the quality of this co-registration, the co-registered T1 image was spatially normalised to the T1 template supplied with SPM8; then, the parameters describing this transformation (*i.e.*, T1 spatial normalisation) were applied to the realigned functional images. Subsequently, these normalised EPIs were spatially smoothed using a Gaussian kernel (6 mm full-width at half maximum). ArtRepair was then used to detect and replace images exhibiting highly variant signal intensity using an interpolation algorithm.

#### 2.6.4. fMRI Modelling: Participant Level Analysis

For each participant, the preprocessed functional images for each dietary condition were modelled in two ways for each of the RVIP and IT tasks separately: (1) all sessions were modelled together to obtain statistical parametric maps that were not biased toward any particular session; and (2) image data were modelled separately for each testing session (three separate models) using the same modelling parameters. In both models, the time-series was first high-pass filtered (150 s) and, then, entered into a multiple regression model. The RVIP data were modelled as a block design, whereas the IT data were modelled as an event-related design.

*RVIP*: each condition (RVIP task and RVIP control) was modelled by box-car regressors defined by the onset and duration of the blocks of stimuli representing each condition separately (*i.e.*, 2 regressors). These regressors were convolved with a canonical hemodynamic response function (HRF) supplied with SPM8. Rest periods were not modelled explicitly and, thereby, contributed to the implicit baseline. To control for fluctuation in the blood oxygen level dependent (BOLD) signal arising from head movement during scanning, the six parameters describing the realignment of each image (3 representing translational and 3 rotational movement) were added to the model as regressors of no interest for each testing session. After estimating the models, contrast maps depicting RVIP Task > RVIP Control were computed.

*IT*: the original trial types (40 ms, 60 ms, 80 ms, 100 ms and 120 ms IT times) were collapsed into correct and incorrect events and modelled by two separate regressors (*i.e.*, correct IT events and incorrect IT events) depicting the onset of such trials by stick functions that were convolved with a canonical HRF supplied with SPM8. The six realignment parameters for this time-series were also added to the model to account for motion-correlated BOLD signal variance. After estimating the models, contrast maps depicting correct IT > incorrect IT were computed.

#### 2.6.5. fMRI Modelling: Participant Level Analysis

The contrast images for the larger model were entered into a one-sample *t*-test. Statistical thresholding for the resultant group activation map was *p* < 0.001 (uncorrected) at the voxel level, and *p* < 0.05 at the cluster-level (family-wise-error corrected for multiple comparisons) was considered significant. The peak voxel of each cluster was used to define regions of interest (ROIs) using the Marsbar Region of Interest toolbox for SPM [36]. Initially, a sphere of 10 mm radius was constructed centred on the peak voxel. The mean contrast estimate for each contrast within each ROI was extracted from each session-specific contrast map yielding three values per ROI for each participant corresponding to Placebo, Berocca Boost and Berocca Performance sessions. These mean ROI values were entered into a repeated-measures ANOVA with treatment (Placebo *vs.* Berocca Performance *vs.* Berocca Boost) as the within-subjects factor in SPSS (Version 19). Due to the small sample size and the exploratory nature of this study, we did not correct the statistical threshold for multiple comparisons.

## 3. Results

### 3.1. Mood and Cognitive Performance

Mood outcomes were analysed by treatment (placebo, Performance, Boost) × time (baseline, post-treatment) × pre-post Cognitive Demand Battery (CDB); summary mood data are presented in Table 2. This revealed significant pre-post effects on three mood measures, indicating that performing the CDB reduced alertness [*F*(1,14) = 34.25, *p* < 0.001] and contentment [*F*(1,14) = 20.34, *p* < 0.001], while increasing mental fatigue [*F*(1,14) = 12.521, *p* = 0.003] and stress [*F*(1,14) = 15.405, *p* = 0.002], all independent of treatment.

**Table 2:** The effects of vitamin-minerals alone (Berocca Performance) and with guaraná (Berocca Boost) on self-rated mood at baseline and post-treatment pre- and post-testing. Means with standard deviations are presented for the three Bond-Lader derived scores and the single visual analogue “stress” and “mental fatigue” scales.

		Baseline		Post-treatment	
Measure	Treatment	Pre	Post	Pre	Post
Bond-Lader Alert	Placebo	66.96 (16.25)	58.90 (18.03)	67.00 (15.26)	62.50 (18.06)
	Berocca Performance	66.77 (12.86)	60.27 (15.46)	66.43 (15.71)	58.61 (16.56)
	Berocca Boost	66.26 (15.09)	58.94 (15.89)	63.98 (13.35)	60.76 (15.90)
Bond-Lader Content	Placebo	70.67 (11.87)	64.93 (15.76)	71.94 (10.98)	68.70 (12.57)
	Berocca Performance	72.51 (9.23)	69.15 (11.69)	72.14 (10.77)	67.28 (13.29)
	Berocca Boost	72.54 (12.29)	66.54 (11.50)	69.46 (11.91)	69.22 (12.32)
Bond-Lader Calm	Placebo	66.85 (14.24)	65.50 (13.61)	62.05 (14.23)	65.50 (13.61)
	Berocca Performance	66.35 (14.40)	64.70 (12.94)	64.80 (15.60)	63.45 (14.00)
	Berocca Boost	65.95 (15.71)	62.43 (10.10)	62.48 (13.81)	61.85 (13.26)
VAS Stress	Placebo	28.05 (19.85)	32.70 (19.84)	28.05 (19.85)	32.70 (19.84)
	Berocca Performance	23.70 (15.38)	32.35 (18.51)	25.45 (16.47)	30.90 (17.07)
	Berocca Boost	27.20 (23.54)	38.45 (21.25)	28.15 (19.20)	33.50 (19.91)
VAS Fatigue	Placebo	31.80 (18.95)	37.35 (22.81)	31.80 (18.95)	32.70 (19.84)
	Berocca Performance	31.85 (16.85)	42.00 (20.12)	33.15 (16.62)	43.40 (20.88)
	Berocca Boost	37.15 (22.79)	45.00 (21.03)	36.15 (15.15)	39.25 (20.93)

For contentment, there was also a significant treatment × pre-post × time interaction [*F*(2,28) = 4.78, *p* = 0.017]. Inspection of the pattern of results suggests that this was due to Boost protecting against the decrease in contentment associated with performing the CDB (Table 2).

**Figure 1:**
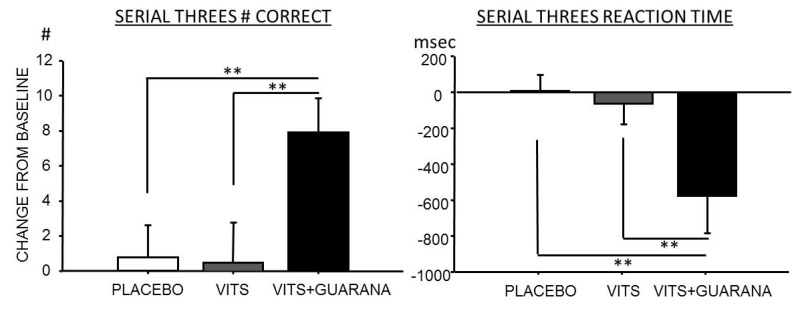
Effects of vitamin-minerals alone (VITS) and with guaraná (VITS+GUARANÁ) on Serial Threes performance compared with a placebo. Graphs depict means with SEM. ** *p* < 0.01.

There was a significant effect of treatment on Serial Threes performance [*F*(2,28) = 6.186, *p* = 0.006]. Pairwise comparisons revealed that this was due to improved performance in the Boost condition compared with both Placebo and Performance (see Figure 1). There were no other significant treatment related mood or cognitive effects.

### 3.2. Brain Activation Associated with the fMRI Tasks

Performing the RVIP task was primarily associated with activation of dorsolateral prefrontal regions, the right superior parietal lobule and the hippocampus (see Table 3a for areas activated). Correct trials during the IT task were primarily associated with activation in the cerebellum and in the left middle temporal gyrus (see Table 3b).

**Table 3:** Areas of BOLD activation during (**a**) the Rapid Visual Information Processing (RVIP) task and (**b**) the Inspection Time (IT) task; L = left, R = right, MNI = Montreal Neurological Institute.

Comparison	L/R	Region	Peak voxel *T*-value	Cluster	Coordinates (MNI) *X*, *Y*, *Z*
*a) RVIP* Task > control	**L**	Superior Parietal Lobule	32.37	830	−30, −66, 52
	**L**	Cerebellum	20.59	847	−28, −72, −26
	**R**	Supplementary Motor Area	22.40	196	4, 8, 54
	**R**	Superior Parietal Lobule	17.89	1032	30, −60, 54
	**L**	Inferior Occipital Gyrus	15.64	204	−28, −88, −10
	**L**	Inferior frontal gyrus	13.81	206	−42, 6, 28
	**L**	Hippocampus	13.33	229	−18, −36, 12
	**L**	Precentral gyrus	12.86	208	−38, −10, 66
	**R**	Middle frontal gyrus	7.74	150	42, 40, 32
*b) IT* Correct > incorrect	**R**	Caudate nucleus	20.90	4253	14, 12, 10
	**R**	Cerebellum	9.32	225	38, −66, −34
	**R**	Cerebellum	9.11	605	8, −54, −42
	**L**	Middle temporal gyrus	9.27	258	−60, −8, −6

### 3.3. Brain Activation Associated with Treatments

**Figure 2:**
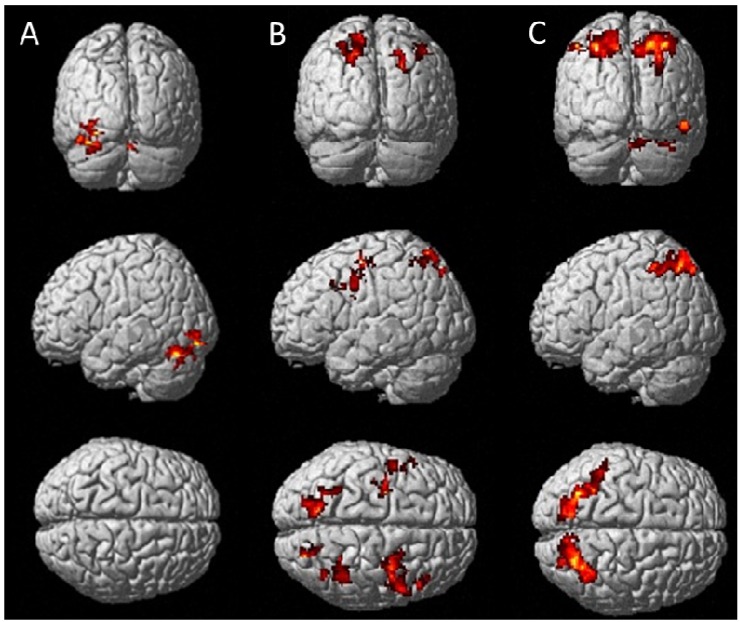
Posterior (**top panel**) and left lateral surface (**middle**) and dorsal (**bottom**) views of brain surfaces showing activation during the RVIP task for Berocca Performance > Placebo (**A**), Berocca Boost > Placebo (**B**) and Berocca Boost > Berocca Performance (**C**).

**Figure 3:**
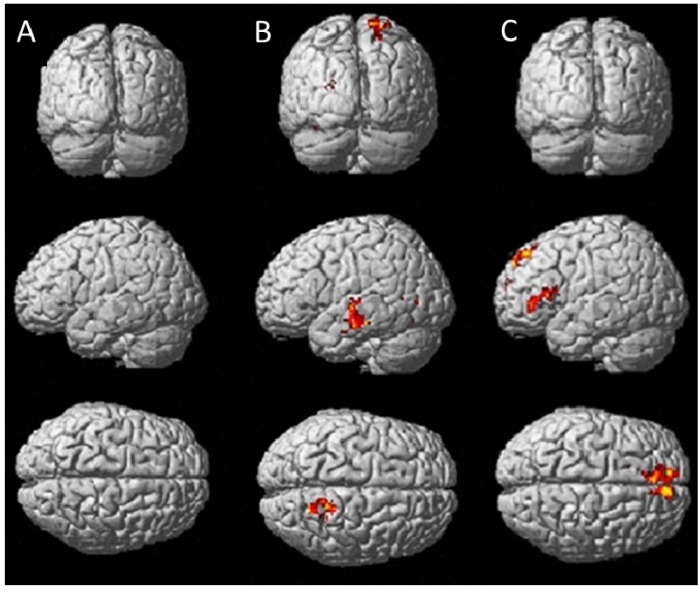
Posterior (**top panel**) and left lateral surface (**middle**) and dorsal (**bottom**) views of brain surfaces showing activation during the IT task for Berocca Performance > Placebo (**A**), Berocca Boost > Placebo (**B**) and Berocca Boost > Berocca Performance (**C**).

Brain activation patterns associated with treatments are presented in Figure 2 (RVIP) and Figure 3 (IT), showing regions of significantly greater activation (*p* < 0.01) of multivitamins without guaraná over placebo (A), multivitamins with guaraná over placebo (B) and multivitamins with guaraná over multivitamins without guaraná (C). 

### 3.4. Brain Activation Associated with Treatments

Brain activation patterns associated with treatments are presented in Figure 2 (RVIP), Figure 3 (IT), showing regions of significantly greater activation (*p* < 0.01) of multivitamins without guaraná over placebo (A), multivitamins with guaraná over placebo (B) and multivitamins with guaraná over multivitamins without guaraná (C).

Whole brain analyses during the RVIP task showed that, compared with placebo, Berocca Performance was associated with increased activation of regions, including the right precentral gyrus and the left and right cerebellum (Figure 2A), while Berocca Boost was associated with greater activation of the right precentral gyrus, the left middle frontal gyrus, frontal medial gyri and the left and right superior parietal lobes. Comparing the effects of Berocca Boost with Berocca Performance, the former was associated with increased activation of both the left and right superior parietal lobe and the right cerebellum (Figure 2C).

Treatment related effects were less pronounced during the IT task with negligible activation differences between Berocca Performance and placebo (Figure 3A), but increased activation of the superior temporal gyrus and superior parietal cortex when comparing Berocca Boost with placebo (Figure 3B). Relative to Berocca Performance, Berocca Boost was associated with increased activation in the left inferior frontal gyrus and (bilaterally) the superior frontal gyrus (Figure 3C).

## 4. Discussion

This study investigated the acute effects of different multivitamin preparations with (Berocca Boost) and without guaraná (Berocca Performance) on mood, cognitive performance and brain activation in healthy young adults. It was predicted that compared with placebo, administration of multivitamin with guaraná would acutely enhance mood and cognitive performance. Consistent with our hypothesis, Berocca Boost improved self-rated contentment and improved (speeded) performance of a working memory/attentional task (Serial Threes) compared with placebo. The effects of the multivitamin with guaraná preparation served as a positive control against which to gauge the effects of the multivitamin without guaraná preparation. There were no acute effects of the latter on mood or cognitive performance, suggesting that the previously reported cognitive enhancement from Berocca Boost [20] may be attributable to its guaraná content.

These beneficial effects of Berocca Boost may be attributable to the well-established positive attentional effects of caffeine and caffeine-like substances contained in guaraná [21]. These behavioural findings are consistent with past research into the neurocognitive effects of guaraná [21]. The guaraná content in the current study was 222 mg, falling squarely within the 37.5 to 300 mg range previously associated with increased self-rated contentment at later time points to here [22]. Additionally, the same multivitamin-guaraná preparation as here was associated with an improvement in RVIP performance (both accuracy and reaction time), coupled with reduced self-rated mental fatigue [20]. In that study, subjects underwent six cycles of the Cognitive Demand Battery (CDB) starting 30 min post-treatment. While the positive effects of Berocca Boost on mental fatigue did not reach significance until the third cycle (50–60 min post dose), treatment was associated with beneficial effects on working memory during the first cycle, 30–40 min post dose. Thus our data showing benefits to attentional/working memory performance (Serial Threes) are largely consistent with the time course of effects of Berocca treatments observed in this previous study. It was not possible to include a guaraná-only condition to definitively determine whether these effects were attributable to caffeine, guaraná or their interaction with other components in Berocca Boost. Using the Cognitive Demand Battery, we have previously shown that caffeine at approximately the 40 mg level used here combined with glucose improves performance [30], albeit targeting RVIP and somewhat later post-treatment. Additionally, an energy drink containing guaraná-derived caffeine was associated with a trend for improved Serial Threes improvement [37]. More work is needed to further disentangle the interactions between guaraná, caffeine and other commonly co-consumed bioactive ingredients. We did not find behavioural benefits from the multivitamin without guaraná treatment. This is unlike one previous report in healthy humans [19], where cognitive performance was improved acutely following multivitamin administration. It is worth noting that there are key methodological differences between that and this study, including: the cohort (children rather than young adults), cognitive tests (a full test battery rather than tests focused on cognitive demand), timing (one and 3 h rather than 30 min post-treatment) and a different multivitamin preparation. Further research is needed to discern which of these factors are important in capturing the acute cognitive effects of multivitamins. 

Unlike the behavioural tests, there was evidence of central activity from both treatments in the small fMRI study, where both preparations were associated with different patterns of activation during in-scanner activation tasks. During the RVIP task, following administration of Berocca Performance, there was greater activity of the cerebellum bilaterally and of the right precentral gyrus compared to placebo. In the context of the RVIP, the right precentral gyrus has been implicated in response inhibition [38]. Regarding the cerebellum, although this structure was once believed to be primarily involved in motor control, recent evidence suggests it has a key role in a variety of cognitive functions, including those involved in successful RVIP performance, such as aspects of attention, executive control and working memory [39,40]. Areas undergoing more activation by Berocca Boost than placebo during the RVIP include, again, the right precentral gyrus, as well as the left middle frontal gyrus, frontal medial gyri and the left and right superior parietal lobes, areas known to be involved in the functional architecture of working memory [41,42]. A more anatomically localised set of working memory related structures (the left and right superior parietal lobe and the right cerebellum) were activated more following Berocca Boost when compared with Berocca Performance.

There was less treatment-related activation during the IT task. Little increased activation was evident when comparing Berocca performance with placebo. Compared with placebo, Berocca Boost administration resulted in a greater activation in the superior temporal gyrus, which plays a role in attentional processing [43], and, again, the superior parietal gyrus, possibly reflecting a working memory component. Comparing Berocca Boost with Berocca Performance revealed greater activation of the left inferior frontal gyrus, known to be involved in inhibition and attentional control [44], and the superior frontal gyri, which may be implicated in visual working memory [45].

While these preliminary neuroimaging findings should not be over-interpreted, they do however provide possible insights into the behavioural effects of the treatments. Berocca Boost was generally associated with greater activation of brain loci involved in working memory and attention, which is consistent with improved Serial Threes performance and which may be attributable to guaraná in the preparation. It is also worth noting that treatment-specific activation changes occurred in the absence of significant in-scanner cognitive effects. This may be due to the small sample size or the tasks being designed primarily to activate the BOLD signal. It is also possible that the changes reflect levels of neural activation that are sub-threshold for behavioural change. Nevertheless, it is striking that a multivitamin preparation (without guaraná) evinced brain activation acutely during the RVIP task. The fact that there was no parallel activation from the same treatment during the IT task gives us more confidence that these effects are not artefacts. Moreover, the crossover design used in this study enabled the comparison of the same individuals under different conditions, thus eliminating inter-individual variance. No adjustment was made for multiple comparisons, although changing the alpha level to account for the number of treatments would not have changed the pattern of significant group differences (all < 0.01). In the case of the behavioural measures, the possibility of Type 1 error was minimised by restricting primary analyses to pre-planned comparisons to those between placebo and each active treatment. Performance following Berocca Boost and Berocca Performance was compared statistically as a secondary analysis only, partly because the vitamin levels of Performance are generally higher than those of Boost (Table 1). This does raise the intriguing possibility that there may be a dose effect of multivitamins and that guaraná combined with a higher level of vitamins may have produced bigger effects. In the case of the fMRI group, this was a small exploratory study. A larger sample size would be needed to better assess the effects of the Berocca treatments on brain functional activation and to generalise these findings to a larger population. Additionally, given that the cognitive effects of multivitamins are more evident with chronic dosing, we are currently embarking on a longer term trial of Berocca Performance incorporating fMRI in a larger cohort. 

The finding that multivitamins (alone) have positive effects on neural and vascular response during higher order cognitive tasks could be investigated further using methodologies assessing changes in cerebral glucose metabolism, blood flow, neurotransmitter levels and levels of brain metabolic by-products using positron emission tomography (PET), magnetic resonance spectroscopy (MRS) and near-infrared spectroscopy (NIRS). 

## 5. Conclusions

In summary, this study shows that multivitamins acutely improve neurocognitive processing. In the case of multivitamins with guaraná, there was increased self-rated contentment and improved attentional/working memory performance, which may be attributable to the activation of working memory/attentional networks as revealed by fMRI. That we also saw increased brain activation from the multivitamin without guaraná treatment suggests that there are acute central effects from multivitamins, which merit further investigation. Hence, our finding suggests that both Berocca Boost and Berocca Performance modify the functional activation of a key brain region underlying high-order cognitive functions. To the best of our knowledge, this is the first fMRI experiment that demonstrates that a multivitamin supplementation with and without guaraná impacts both neural activity and cognition.
